# Hyperhomocysteinemia in women with unexplained sterility or recurrent early pregnancy loss from Southern Italy: a preliminary report

**DOI:** 10.1186/1477-9560-5-10

**Published:** 2007-07-11

**Authors:** Maristella D'Uva, Pierpaolo Di Micco, Ida Strina, Carlo Alviggi, Mariateresa Iannuzzo, Antonio Ranieri, Antonio Mollo, Giuseppe De Placido

**Affiliations:** 1 Dipartimento di Scienze Ostetriche Ginecologiche e Medicina della Riproduzione, Area Funzionale di Medicina della Riproduzione ed Endoscopia Ginecologica, University of Naples "Federico II", Italy; 2 Internal Medicine Division, Fatebenefratelli Hospital of Naples, Naples, Italy

## Abstract

**Background:**

Hyperhomocysteinemia has been described as a risk factor for unexplained recurrent pregnancy loss. Increased levels of homocysteine may be due to inadequate dietary intake of folate and vitamin B12 and inherited defects within the methionine-homocysteine pathway such as MTHFR C677T gene polymorphism. However, the association between hyperhomocysteinemia and sterility problems have been underlined only for recurrent pregnancy loss while a relationship between hyperhomocysteinemia and female sterility is still matter of discussion.

**Aim:**

This study sought to find out a possible relationship between sterility (primary sterility or secondary sterility due to recurrent pregnancy loss) and homocysteine metabolism.

**Patients and Methods:**

We selected 20 patients with recurrent pregnancy loss, 20 patients with unexplained female sterility and 20 healthy women as control group. Several whole blood samples were collected by venipuncture. Firstly homocysteinemia and other related variables were tested (i.e. folate and vitamin B12 levels); thereafter DNA was extracted by a further whole blood sample collected in EDTA in order to screen MTHFR C677T gene polymorphism. Statistical analysis was performed by chi square test; differences were considered to be significant if p < 0.05.

**Results:**

The median fasting total plasma homocysteine concentration was 19.2 ± 6.14 μM for patients with recurrent pregnancy loss, while was 21.05 ± 8.78 μM for patients with unexplained sterility, vs 7.85 ± 3.31 μM of control group (p < 0.05). Fifteen patients with unexplained female sterility showed MTHFR C677T homozigosity vs 17 with recurrent pregnancy loss and 3 in the control group (p < 0.05). On the other hand no significant differences were found in the levels of vitamin B 12 in the three groups, while reduced folate concentrations were found in women with unexplained female sterility and recurrent pregnancy loss (p < 0.05 vs control group.

**Discussion:**

MTHFR C677T gene polymorphism is frequent in the studied populations. These data raise questions on the role of the homocysteine metabolism in sterility problems. Even though increased homocysteine (i.e. > 15 μM) and MTHFR C677T homozigosity have already been described as risk factors for recurrent pregnancy loss, few studies evaluated their role in women with unexplained sterility. Further studies on larger series are needed to better understand the role of homocysteine metabolism, including folate metabolism, in this clinical setting.

## Background

Hyperhomocysteinemia (HHCY) has been underlined as an emerging risk factor for several diseases such as arterial and/or venous thrombosis [1], adverse pregnancy outcome [2,3], congenital malformations [4] and vascular dementia [5,6]. Inherited and acquired conditions have been involved to explain pathophysiology of HHCY such as gene polymorphisms [i.e. cystationin beta synthase (CBS) or methylenetetrahydrofolate reductase (MTHFR)] [7,8] and folate and/or vitamin B6/B12 deficiencies due to disregulation of their normal metabolism and/or low dietary intake [9-11]. Because HHCY has frequently been associated to clinical vascular thrombosis, homocysteine metabolism is investigated, together with other thrombophilic conditions such as inherited and/or acquired thrombophilia, in patients with early onset of vascular thrombosis [1]. However, in this field inherited and/or acquired thrombophilia are identified as well known risk factors for adverse pregnancy outcome, in particular in case of early recurrent pregnancy loss (RPL). Literature already underlined a strong relationship between inherited deficiency of protein C, protein S or antithrombin and RPL [12]. Moreover, gene variants related to thrombophilia as factor V Leiden and\or prothrombin A20210G have been reported to be associated to RPL [13-15]. Moreover, acquired conditions such as antiphopspholipid syndrome or increased plasma levels of clotting factor VIII have been associated to RPL [16-19]. Furthermore, increasing evidence is now available also for the association of HHCY and RPL [2,3].

Previous studies failed to include or exclude other causes of miscarriages, when alteration of haemostasis with a trend toward hypercoagulable state were considered. On the other hand, few emerging data are now available for the role of inherited and/or acquired thrombophilia in women with in vitro fertilisation failure [20,21], while data about the association of female sterility and alteration of haemostasis are lacking, in particular if the homocysteine metabolism is considered.

The aim of our study was therefore to investigate the role of homocysteine metabolism in patients with unexplained female sterility or secondary sterility due to RPL.

## Patients and Methods

### Patients selection

We observed 125 consecutive women referred to our Sterility Center for infertility due to RPL or for female sterility. We considered for this study in the group of women with RPL all patients with 2 or more first trimester abortion or with 1 or more late pregnancy loss, while we considered women without any clear evidence of pregnancy in their anamnesis in the group of women with unexplained female sterility (UFS).

In order to evaluate the causes of RPL or female sterility we looked for chromosomal alterations, endocrine dysfunctions, chronic inflammatory diseases, infectious diseases, uterine malformations, tubal patency, alteration of haemostasis with a trend toward thrombophilia.

All patients underwent karyotype study in order to detect several chromosomal aberrations such as balanced translocations.

Anatomic evaluation of the uterine cavity was performed by transvaginal ultrasound scan and hysterosalpingography and/or hysteroscopy in order to detect mullerian malformations or the presence of fibroids or polyps and\or tubal patency.

Endocrinological assessment included screening for diabetes, hypothyroidism, hypopituitarism, hyperprolactinaemia, luteal insufficiency and polycystic ovarian syndrome (PCOS). Basal FSH, LH and oestradiol, luteal phase progesterone, TSH, prolactin levels and fasting glucose were evaluated in all patients. In addition, trans-vaginal USG and androgens levels were assessed to look for PCOS and\or anovulation.

Chronic inflammations due immunological diseases such as systemic erythematosus lupus, rheumatoid arthritis, systemic sclerosis were also studied. Serum level of antinuclear antibodies (ANA), antimithocondrial antibodies (AMA), smooth muscle antibodies (SMA), rheumatoid factor and levels of C reactive protein were assessed. Infective disease due to *Chlamydia spp*, were evaluated by specific *Chlamydia *assays (i.e. serological levels of specific IgG and IgM against *Chlamydia spp*). Moreover, all patients were examined for vaginal and cervical smears, in order to exclude vaginal and\or cervical infections such as *Chlamidia spp or Mycoplasma spp *or mycosis.

Alterations of haemostasis with a trend toward thrombophilia were excluded by specific assays to test protein C, protein S and antithrombin deficiency, anticardiolipin IgG and IgM antibodies, lupus anticoagulant, inherited gene polymorphisms of factor V Leiden and A20210G prothrombin. So, patients carrying hypercoagulable state due the reported variables were excluded from the study.

Moreover, obese patients were excluded, in particular women with Body Mass Index > 25 were not enrolled in the study.

### Patients group

After this screening we selected 20 patients with unexplained RPL and 20 women with UFS.

Selected women were tested for several variables of homocysteine metabolism through methylene-tetra-hydrofolate reductase (MTHFR) C677T gene polymorphism, homocysteinemia, folate and vitamin B 12 levels. All selected patients, both affected by UFS and RPL, were taking preconceptional doses of folic acid (i.e. 400 μg) in order to prevent neural tube defects in case of pregnancy. On the other hand none of control subjects was taking folic acid.

## Methods

Whole blood samples were collected from all selected subjects in the study by venipuncture from antecubital vein in order to screen possible involvement of alteration of homocysteine metabolism. All subjects were assayed for plasma homocysteine (t-Hcy), vitamin B12 and folic acid levels and MTHFR C677T gene polymorphism.

### First blood sample

The first blood sample was collected in EDTA to screen fasting homocysteine (FPIA-Abbott).

### Second blood sample

The second blood sample was collected in SST II advanced tube in order to detect serum folic acid levels and serum vitamin B 12 levels (CMIA-Abbott)

### Third blood sample

A further blood sample (5 mL) was collected in EDTA in order to screen gene variants of MTHFR C677T. DNA was extracted using the "NUCLEON BACC" kit (Amershan, Germany). Patients were screened for the C677T gene polymorphism of MTHFR using PCR amplification with specific primers and the Light Cycler apparatus (Roche, Italy).

### Control group

Twenty age and sex matched healthy subjects were enrolled as control group. Selected subjects had the same ethnical background of the study group. In the control group we included women with one or more successful pregnancy and without gestational complication (intrauterine growth restriction, stillbirth and abruptio placentae) and any abortion. We excluded subjects with previous arterial and/or venous thrombosis. We also excluded subjects with first degree relatives with arterial and/or venous thrombosis before than 65 years old.

### Statistical analysis

Statistical analysis was based on chi square test, differences were considered to be significant if p < 0.05. Statistical analysis was carried out using SPSS statistical software [22,23].

## Results

Fasting levels of homocysteinemia were higher both in patients with UFS (i.e. 21.05 ± 8.78 μM) and with RPL (19.20 ± 6.14 μM) compared to control subjects (7.85 ± 3.31 μM); differences were both statistically significant (p: <0.01) (Table 1 and 2). Fasting homocysteine was slightly increased in patients with unexplained sterility compared to patients with RPL, but this difference did not reach statistical significance (Table 1 and 2).

**Table 1:** Data of homocysteine metabolism in patients with sterility or RPL and in control subjects

**Test (unit of measurement)**	**UFS (subjects 20)**	**RPL (subjects 20)**	**CG (subjects 20)**
*Hcy (μM/ml)*	21.05 ± 8.78	19.20 ± 6.14	7.85 ± 3.31
*Serum folic acid (ng/ml)*	6.70 ± 4.50	6.10 ± 2.81	20.10 ± 9.44
*Serum vitamin B 12 (pg/ml)*	648 ± 162	608 ± 154	664 ± 175
*MTHFR C677T heterozigosity*	5/20	3/20	9/20
*MTHFR C677T homozigosity*	15/20	17/20	3/20

Hcy: homocysteine

MTHFR: methylenetetrahydrofolate reductase

UFS: unexplained female sterility group

RPL: recurrent pregnancy loss group

CG: control group

**Table 2:** Statistical differences between patients with unexplained female sterility, patients with RPL and control subjects

	**Hcy (μM/ml)**	**p**	**Serum folate (ng/ml)**	**P**	**Serum vit. B 12 (pg/ml)**	**p**	**MTHFR C677T heterozigosity**	**p**	**MTHFR C677T homozigosity**	**p**
*UFS vs CG*	21.05 ± 8.78 vs 7.85 ± 3.31	<0.01, s	6.70 ± 4.50 vs 20.10 ± 9.44	<0.01, s	648 ± 162 vs 664 ± 175	0.35, ns	5/20 vs 9/20	0.32, ns	15/20 vs 3/20	<0.01, s
*UFS vs RPL*	21.05 ± 8.78 vs 19.20 ± 6.14	0.44, ns	6.70 ± 4.50 vs 6.10 ± 2.81	0.007, s	648 ± 162 vs 608 ± 154	0.42, ns	5/20 vs 3/20	0.69, ns	15/20 vs 17/20	0.69, ns
*RPL vs CG*	19.20 ± 6.14 vs 7.85 ± 3.31	<0.01, s	6.10 ± 2.81 vs 20.10 ± 9.44	<0.01, s	608 ± 154 vs vs 664 ± 175	0.20, ns	3/20 vs 9/20	0.08, ns	17/20 vs 3/20	<0.01, s

Hcy: homocysteine

MTHFR: methylenetetrahydrofolate reductase

UFS: unexplained female sterility group

RPL: recurrent pregnancy loss group

CG: control group

ns: not significant

s: significant

Moreover, serum folic acid levels were lower both in women with UFS (6.70 ± 4.50 ng/ml) and by RPL (6.10 ± 2.81 ng/ml) compared to control subjects (20.10 ± 9.44 ng/ml) and also these differences reached statistical significance (p: <0.01) (Table 1 and 2). Interestingly, in this case we found also a significant statistical difference in folate concentrations in women with unexplained sterility compared to women with RPL (p: 0.007) (Table 1 and 2).

Serum vitamin B12 levels were comparable in women with UFS (648 ± 162 pg/dl), in women with RPL (608 ± 154 pg/dl) and in control subjects (664 ± 175 pg/dl) (Table 1 and 2) not reaching significant statistical differences (Table 2).

MTHFR C677T gene polymorphism was searched for in all subjects with UFS, RPL and in controls. Five/20 patients with UFS and 3/20 patients with RPL showed heterozigosity for MTHFR C677T compared to 9/20 of control group (Table 2).

MTHFR C677T homozigosity was present in 15/20 patients with UFS and 17/20 patients with RPL compared to 3/20 subjects of control group (Table 1); differences were significant both for sterility and RPL when compared to control group (p: <0.01 UFS vs control group and RPL vs control group) (Table 2). No differences were found between UFS and RPL (p: 0.69, ns) (Table 2). None of the subjects with UFS or RPL were wild type for MTFHR C677T gene polymorphism vs 8\20 subjects of control group.

Results showing variables in patients with unexplained female sterility, RPL and control group are summarised in Table 1 and 2.

## Discussion

Since 1990 several studies underlined a pathogenic role for inherited thrombophilia in women with RPL [24,25]. A pathogenic role was identified for inherited deficiency of protein C, protein S and antithrombin and for inherited gene polymorphism of factor V Leiden and A20210G of prothrombin, and also for acquired thrombophilia, in particular antiphospholipid syndrome [12-19].

Also a potential pathogenic role of HHCY has been recently suggested, because the association of HHCY and thrombosis, but not univocal data are available. Increasing evidences are available for the relationship between HHCY and MTHFR C677T gene polymorphism and unexplained recurrent pregnancy loss. Several reports, in fact, described an association between early RPL and HHCY and/or MTHFR C677T gene polymorphism [2,3,26,27]. A different point of view on the association between HHCY and RPL has been reported only by Makino et al. [28].

Only a few studies are available on the association between sterility and HHCY and they are focused only on women with in vitro fertilization failures. Moreover, available data seem to be in contrast: Martinelli et al. did not find an association between inherited thrombophilia and patients with in vitro fertilization failure [29], while Azem et al. and Qublan et al. underlined a possible role of inherited thrombophilia and IVF failure [20,21]. However, both studies were not based on a possible association of homocysteine metabolism and IVF failure but only on the C677T gene polymorphism of MTHFR.

In the present study we evaluated not only homocysteinemia and MTHFR C677T gene polymorphism but also other common variables associated with homocysteine metabolism such as folate and vitamin B12 serum levels both in women showing RPL and women with UFS.

We found that women with RPL and UFS showed HHCY compared to control group (Table 1 and 2). We did not find differences in homocysteine levels between women with RPL and women with UFS. Although data concerning HHCY and RPL seem to be in agreement with those already reported [2,3,26,27], data focusing the association between of HHCY and UFS are innovative because rarely described so far.

Data concerning MTHFR C677T gene polymorphism seem to support this hypothesis because we found a high frequency of homozigosity of TT genotype not only in women with RPL but also in women with UFS (Table 1). TT genotype of MTHFR C677T gene polymorphism has been already underlined in pathophysiology of RPL by several Authors but its role in pathophysiology of UFS is still a matter of discussion. Therefore, our data support the hypothesis concerning the involvement of homocysteine metabolism in cases of UFS because frequency of TT genotype was similar to that observed in women with RPL (Table 2). Moreover, our data may also support previous observations of an increased frequency of thrombophilia in women with infertility showing repeated IVF failures.

Serum folate and vitamin B12 levels were also tested to support this hypothesis because strongly associated to homocysteine metabolism, also in terms of a possible therapeutic support. Although, vitamin B12 levels were lower both in women with RPL and with UFS compared to control subjects, and these differences did not reach statistical significance (Table 1 and 2). On the other hand we found significantly lower folate levels both in women with RPL and UFS when compared with control subjects (Table 1 and 2). These data confirm the strict association between folate metabolism and homocysteine levels also in pathophysiology of women with infertility, in particular if referred to adverse pregnancy outcome such as early RPL. Moreover, for the first time we underlined a possible association between low serum folate and HHCY and UFS. Furthermore, from another point of view our data offer a new scenario on the possible therapeutic support with folic acid fortification both in women with RPL and UFS carrying HHCY.

In conclusion our study provides several data concerning the involvement of homocysteine metabolism in women with infertility: we confirmed a strict association between HHCY and TT genotype of MTHFR C677T in women with RPL without other causes of recurrent abortion. Yet, for the first time, we suggested also that homocysteine metabolism may be involved in pathophysiology of these cases of UFS because of the association between HHCY, low serum folate and TT genotype of MTHFR C677T. However, because of the small number of selected patients, our data should be confirmed by further studies based on larger population.
